# Worse Physical Disability Is Associated With the Expression of PD-1 on Inflammatory T-Cells in Multiple Sclerosis Patients With Older Appearing Brains

**DOI:** 10.3389/fneur.2021.801097

**Published:** 2022-01-06

**Authors:** Sophie A. H. Jacobs, Paolo A. Muraro, Maria T. Cencioni, Sarah Knowles, James H. Cole, Richard Nicholas

**Affiliations:** ^1^Department of Computer Science, Centre for Medical Image Computing, University College London, London, United Kingdom; ^2^Department of Neurology, Imperial College Healthcare, London, United Kingdom; ^3^Division of Clinical Neurology, Department of Brain Sciences, Imperial College London, London, United Kingdom

**Keywords:** multiple sclerosis, brain age, biomarkers, immunology, MRI, atrophy

## Abstract

**Background:** Magnetic Resonance Imaging (MRI) analysis method “brain-age” paradigm could offer an intuitive prognostic metric (brain-predicted age difference: brain-PAD) for disability in Multiple Sclerosis (MS), reflecting structural brain health adjusted for aging. Equally, cellular senescence has been reported in MS using T-cell biomarker CD8^+^CD57^+^.

**Objective:** Here we explored links between MRI-derived brain-age and blood-derived cellular senescence. We examined the value of combining brain-PAD with CD8^+^CD57^+^(ILT2^+^PD-1^+^) T-cells when predicting disability score in MS and considered whether age-related biological mechanisms drive disability.

**Methods:** Brain-age analysis was applied to T1-weighted MRI images. Disability was assessed and peripheral blood was examined for CD8^+^CD57^+^ T-cell phenotypes. Linear regression models were used, adjusted for sex, age and normalized brain volume.

**Results:** We included 179 mainly relapsing-remitting MS patients. A high brain-PAD was associated with high physical disability (mean brain-PAD = +6.54 [5.12–7.95]). CD8^+^CD57^+^(ILT2^+^PD-1^+^) T-cell frequency was neither associated with disability nor with brain-PAD. Physical disability was predicted by the interaction between brain-PAD and CD8^+^CD57^+^ILT2^+^PD-1^+^ T-cell frequency (*AR*^2^ = 0.196), yet without improvement compared to brain-PAD alone (*AR*^2^ = 0.206; AICc = 1.8).

**Conclusion:** Higher frequency of CD8^+^CD57^+^ILT2^+^PD-1^+^ T-cells in the peripheral blood in patients with an older appearing brain was associated with worse disability scores, suggesting a role of these cells in the development of disability in MS patients with poorer brain health.

## Introduction

Magnetic Resonance Imaging (MRI) has played a pivotal role in multiple sclerosis (MS) diagnostics and disease course predictions (1). However, the relationships between imaging markers and clinical outcome are inconsistent and predicting disease course remains challenging. Recent research showed the promising prognostic value of the so-called “brain age” paradigm in MS (2–4). Brain age uses machine learning to analyze structural MRI data and models healthy aging using a large independent “training” dataset of healthy adults, resulting in a robust biomarker based on neuroimaging measures of atrophy (5). Brain-predicted age difference (brain age—chronological age; brain-PAD) is a simple metric for structural brain health adjusted for brain changes due to healthy brain aging, in which a higher brain-PAD reflects lower brain volumes and thus poorer brain health (5).

In a large multi-center study, Cole et al. (3) found that MS patients had an elevated brain-PAD of 10.3 years, which was positively correlated with higher disability score at study assessment. Longitudinally, an increase in brain-PAD was correlated with worsened disability and it predicted disability progression over the course of 3.4 years (3). However, normalized brain volume (NBV) was a better predictor of disability progression, indicating that brain-PAD is a useful biomarker but not suitable to replace conventional prognosis methods (3). To improve the performance of brain-PAD as a prognostic biomarker, earlier research combined brain-PAD with other aging-related molecular biomarkers (6, 7). For example, Cole et al. combined information on DNA-methylation patterns and telomere length with brain-PAD in order to predict mortality risk (6). While telomere length could not predict mortality risk either alone or in combination with brain-PAD, information on DNA-methylation patterns (the epigenetic “clock”) together with brain-PAD outperformed the predictive value of brain-PAD alone. Seemingly, combining brain-PAD with other aging-related biomarkers could be a promising prognostic tool, which may be relevant to MS (6, 8).

During healthy aging, systematic low-grade chronic inflammation is known to develop, whereby older age is associated with changes in phenotype and functioning of inflammatory markers, a process termed “inflamm-aging” (9). For example, an older age is associated with accumulation of CD8^+^CD57^+^ T-cells (10, 11). These senescent CD8^+^CD57^+^ T-cells are powerful releasers of cytotoxic granules, and modulated by the activation of the inhibitory receptors programmed death 1 (PD-1) and immunoglobulin-like transcript 2 (ILT2) (12, 13). In MS, Cencioni et al. found an increase in PD-1 expressing CD8^+^CD57^+^ T-cells in peripheral blood (PB) during clinical remission but not relapse (13). A weak linear correlation between PD-1 and ILT2 was found, suggesting independent functioning of these two receptors. The importance of PD-1 expression on CD8^+^CD57^+^ T-cells was substantiated by examining post-mortem material of fast-progressing MS patients where a high level of CD8^+^CD57^+^PD-1^+^ T-cells was found in the inflamed meninges, suggesting an involvement of these inflammatory cells in MS progression (13).

Combining neuroimaging-derived brain age with blood-derived CD8^+^CD57^+^ T-cell levels could potentially help improve prognostics in MS (7). Moreover, it might shed light on the biological mechanisms that drive disease progression in MS and how these mechanisms relate to the biology of aging. Here, we studied the utility of combining MRI brain age with measures of CD8^+^CD57^+^(ILT2^+^PD-1^+^) T-cells in predicting disability in a predominantly relapsing-remitting MS (RRMS) patient cohort (*n* = 184, 19–65). The following hypotheses were tested: (i) a greater brain-PAD is associated with a higher Expanded Disability Status Scale (EDSS) score and a higher CD8^+^CD57^+^(ILT2^+^PD-1^+^) T-cell frequency in PB; (ii) a higher EDSS-score is associated with a higher CD8^+^CD57^+^ILT2^+^PD-1^+^ T-cell frequency; and (iii) a higher CD8^+^CD57^+^ILT2^+^PD-1^+^ T-cell frequency combined with a higher brain-PAD is associated with a higher EDSS-score.

## Materials and Methods

### Participants

This observational study used routinely collected data of 184 patients (aged 18–63), collected between 2010 and 2012 by the Patient Research Cohort Rapidly Evolving Multiple Sclerosis Study (PRC-REMS) at Imperial College London. Data collection was conducted in accordance with the principles of Good Clinical Practice (GCP), in approval of the London-Chelsea Regional Ethics Committee (NCT01044576). All patients were highly active and/or had treatment-refractory MS activity, and were diagnosed with predominantly RRMS according to the revised 2005 McDonald Criteria (14). A small minority of patients suffered from secondary progressive MS (*n* = 6) or primary progressive MS (*n* = 1). Exclusion criteria were pregnancy, renal impairments, claustrophobia, diagnosed > 15 years ago, and a Kurtzke EDSS-score > 6.

Patients underwent a T1-weighted MRI brain scan, blood sampling and various tests were done to assess physical disability and cognition. Blood samples were obtained before any corticosteroid therapy. The patients' disease phase was categorized as “active” if they suffered from 2 or more clinical relapses in the 12 months prior to enrolment and if they presented gadolinium-enhancing lesions on the MRI. Patients were other categorized as “stable.” Treatment type was recorded at study enrolment. Some of the patients participating in this study were included in two previous studies (15, 16). Prior to data collection, all participants provided written informed consent.

### Physical and Cognitive Testing

The EDSS was used to evaluate functional systems (17); the 25- foot walk test (25-FWT) measured lower extremities functioning; and the nine-hole peg test (9-HPT) measured functioning of the upper extremities. Reciprocal score of the 9-HPT was used in the analysis in which a smaller reciprocal score represented worse functioning of the upper extremities. Furthermore, cognitive performance was obtained using the paced auditory serial addition test (PASAT), assessing processing speed, calculation abilities and flexibility (18). A higher PASAT-score reflects better cognitive functioning.

### Blood Sampling

After venous puncture, PB mononuclear cells from blood samples were separated according to standard protocols by density gradient over Ficoll-Hypaque (Pharmacia, Uppsala, Sweden) (19). Subsequently, surface staining was performed using anti-human antibodies: CD57, CD8, PD-1, IL-T2, (BD Biosciences, UK) according to previous described protocols (13, 20). Then, the cells were acquired to FACScalibur flow cytometer (BD UK Limited, Berkshire, UK) and FCS files were analyzed with FlowJo software (FlowJo, LLC).

### Magnetic Resonance Imaging Acquisition

Scans were acquired on a single scanner using a 3T clinical MR system (Magnetom Verio, Siemens Medical Solutions, Erlangen, Germany). For reception, a 12-channel phased head coil system was used. T1-weighted 3-dimensional (3D) Magnetization Prepared Rapid Gradient Echo (MP-RAGE) images of the brain were obtained. MRI acquisition was done similarly to the protocol guidelines established in the Alzheimer's Disease Neuroimaging Initiative (ADNI) study (21). Only few parameters were deviating: a 1 mm isotropic resolution and 160 sagittal sections in a single 3D slab was used, with an echo time (TE) of 366 ms; a repetition time (TR) of 3,000 ms; a parallel imaging (PI) factor of 2 and 256 x 192 mm field of view (FOV) in 5 m:21 s. For more detailed information about the MRI acquisition settings, see Lema et al. (15).

### Brain-Predicted Age Difference Analysis

Similar to a previously established protocol, biological brain age was calculated using brainageR software (version 2.1, October 2019; https://github.com/james-cole/brainageR) (5, 22). One hundred and eighty four structural T1-weighted images were pre-processed using SPM12 software (www.fil.ion.ucl.ac.uk/spm/software/spm12/) generating GM, WM and CSF segmentations. Five participants were excluded after visual quality control due to inaccurate segmentation. Then, non-linear registration of the segmented GM, WM and CSF images of the remaining 179 patients was done using a custom template created based on a predefined healthy training dataset. Finally, using SPM-DARTEL, images were registered to Montreal Neurological Institute (MNI) 152 space (voxel size = 1.5 mm^3^), modulated and smoothed (4 mm). Summary volumetric measures of GM, WM, CSF and intracranial volume (ICV) were generated. Then, a predefined machine-learning model of healthy brain aging (independent dataset of *n* = 3,377 healthy individuals, aged 18–92 years, screened for comorbidities) was used, based on principal components analysis (PCA) of processed volumetric neuroimaging data, retaining 80% of the variance. To generate biological brain-predicted ages, PCA loadings were used to weight to the current processed neuroimaging data before the coefficients of the trained model were applied to give a brain-predicted age per participant. Brain-PAD was calculated by subtracting chronological age from brain-predicted age. No automatic correction for a statistical dependency of brain-PAD on chronological age was done.

### Statistical Analysis

Statistical analyses were performed using R version 4.0.3. Pearson's (*r*) or Spearman's (*r*_*s*_) correlation was done between the clinical and demographical variables. Multiple-comparison correction was done using the false discovery rate (FDR). Multiple linear regression models were used to investigate the relationship of brain-PAD with clinical outcomes and potential blood markers in MS. The contribution of these models to the variance seen in the outcome variable is shown by the *R*^2^, where a *R*^2^ of 1.0 represents 100%. To correct for the amount of predictors used in the models, the adjusted *R*^2^ *(AR*^2^*)* was used. Additionally, the standardized beta coefficient (β*)* is used to assess the independent influence of the predictor variables on the outcome variable. Every unit increase in the predictor variable, the outcome variable will increase or decrease by the value of the beta coefficient. Differences between disease phases and treatment groups were analyzed with an ANOVA or its non-parametric equivalent the Kruskal-Wallis test. Multicollinearity was tested, and standardized residuals were checked for normality. To establish whether brain volume measurements were driving variability in brain-PAD, a linear regression with hierarchical partitioning of variance was performed with age, sex, and the distinct brain volumes as predictors. The best model performance was reviewed using Akaike information criterion (AIC) modeling corrected for small sample sizes (AICc).

## Results

### Population Characteristics

Demographics and clinical characteristics of the PRC-REMS cohort are shown in Table 1. Only patients diagnosed within the last 15 years were included in this study, though some suffered from symptoms before clinical diagnosis and therefore have a longer disease duration as shown in Table 1.

**Table 1:** Demographic and clinical characteristics patient cohort.

	**Multiple sclerosis patients**
**Demographic characteristics**	
N	179
Female, n (%)	136 (76%)
Age (years), mean (SD) [range]	38.12 ± 8.37 [18–63]
Age at onset (years), mean (SD) [range]	33.66 ± 8.51 [16–63]
Disease duration (years)	
Mean (SD)	4.58 ± 4.13
Median [range]	3 [0–21]
Patients on DMT, n (%)	
Yes	68 (38.0%)
No	57 (31.8%)
Unknown	54 (30.2%)
**Clinical evaluation**	
MS classification, n (%)	
RRMS	172 (96.1%)
PPMS	1 (0.6%)
SPMS	6 (3.3%)
EDSS, median [range]	3 [1.5–6]
Disease phase, n (%)	
Stable	120 (67.0%)
Active	38 (21.3%)
Unknown	21 (11.7%)
Treatment type, n (%)	
Off treatment	99 (55.3%)
Interferon-beta	49 (27.4%)
Glatiramer acetate	25 (14.0%)
Tysabri	6 (3.3%)

*SD, standard deviation; DMT, disease modifying treatments, use on moment of blood sampling; MS, multiple sclerosis; RRMS, relapsing-remitting multiple sclerosis; PPMS, primary progressive multiple sclerosis; SPMS, secondary progressive multiple sclerosis; EDSS, expanded disability scale status at study assessment; Active Disease Phase, enhanced lesions are present and the patient suffered ≥ 2 Relapses in the year before study enrolment; Unknown Disease Phase, the amount of relapses in the past year is unknown and/or the presence of enhanced lesions is unknown; Stable Disease Phase, the amount of relapses in the past year is <2 and/or enhanced lesions are not present. Treatment Type was registered at the start of study enrolment*.

### Higher EDSS-Score is Associated With Higher Age at Onset, Longer Disease Duration, Higher Chronological Age at Scan and Lower Cognitive Performance

**Figure 1:**
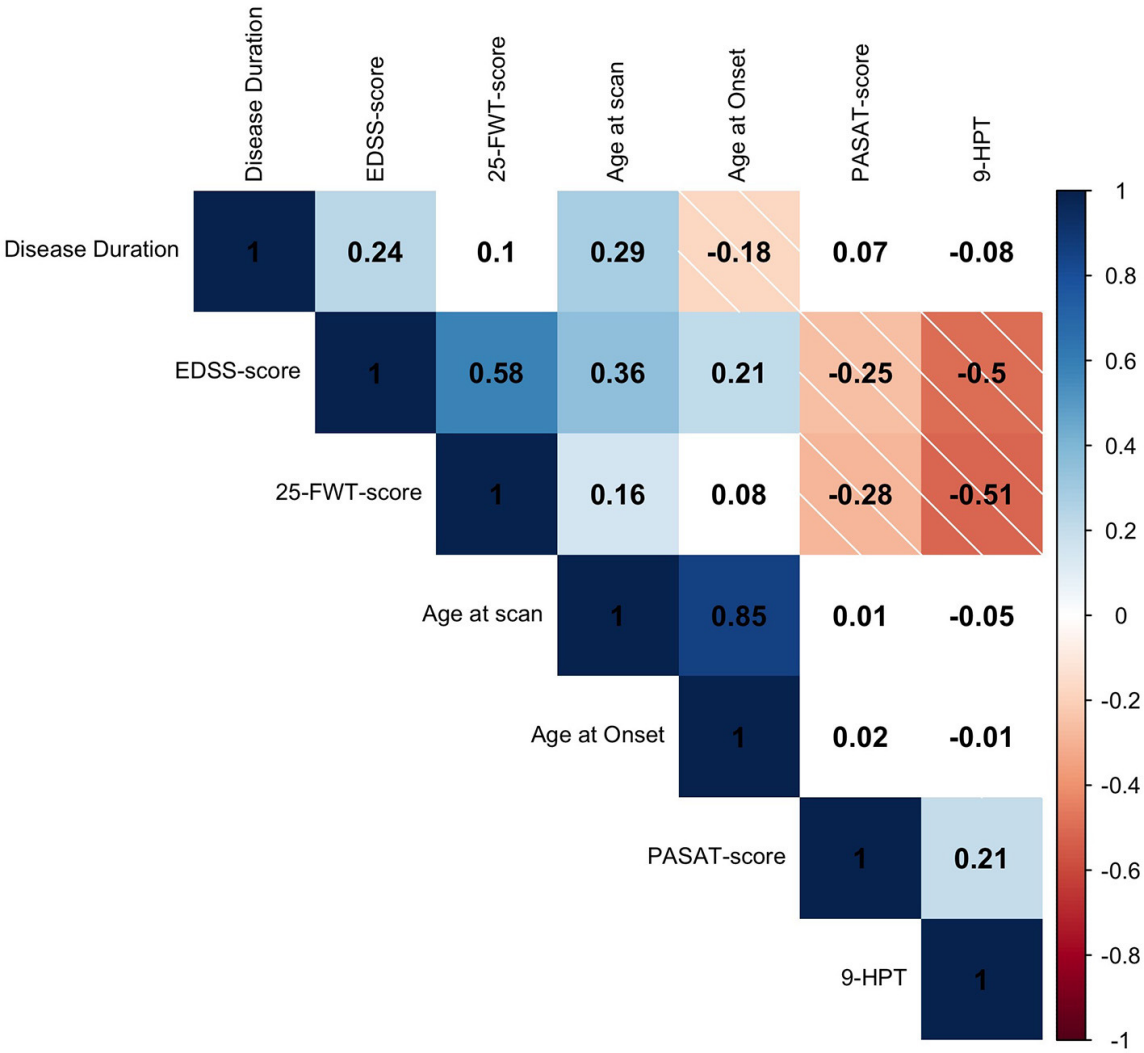
Spearman's rho *(r*_*s*_*)* correlation matrix of clinical variables. Positive correlations are depicted in solid blue and negative correlations are depicted in striped red. The color intensity is proportional to the correlation coefficient. White fields are displaying non-significant relationships (*p* > 0.05). The *r*_*s*_ is given in every field. EDSS, expanded disability scale status; 25-FWT, 25-food walk test; PASAT, paced auditory serial addition test; 9-HPT, reciprocal score of 9-hole peg test.

Higher age at scan was correlated with higher age at MS onset (*r*_*s*_ = 0.85, *p* < 0.001) and longer disease duration *r*_*s*_ = 0.29, *p* < 0.001). Higher EDSS-score at scan was correlated with higher age at scan (*r*_*s*_ = 0.36, *p* < 0.001); longer disease duration *(r*_*s*_ = 0.24, *p* = 0.001); and higher age at onset (*r*_*s*_ = 0.21, *p* = 0.006). Furthermore, higher EDSS-score at screening was correlated with lower PASAT-score (*r*_*s*_ = −0.25, *p* = 0.001), showing that higher physical disability was associated with lower cognitive functioning. The PASAT-score did not correlate with screening age, age at onset or disease duration (*p* > 0.05). Higher 25-FWT-score was correlated with lower PASAT-score (*r*_*s*_ = −0.28, *p* < 0.001); higher EDSS-score (*r*_s_ = 0.58, *p* < 0.001); and higher 9-HPT-score (*r*_*s*_ = −0.51, *p* < 0.001). Worse performance on the 25-FWT was correlated with higher age at scan (*r*_s_ = 0.157, *p* = 0.048), but not with age at MS onset or disease duration (*p* > 0.05). Furthermore, lower performance of the upper extremities (9-HPT) was correlated with higher EDSS-score (*r*_*s*_= −0.50, *p* < 0.001) and worse cognitive performance measured by the PASAT test (PASAT, *r*_*s*_ = 0.21, *p* = 0.01). The 9-HPT was not correlated with either age at MS onset, age at scan nor with disease duration (*p* > 0.05) (Figure 1). Neither treatment type nor disease phase were related to EDSS-score.

### Worse Physical Disability is Associated With Older Appearing Brains in MS

The average brain-PAD in the mainly RRMS patient group was 6.54 [95% confidence intervals (CI): 5.12, 7.95] (RRMS *n* = 172, SPMS *n* = 6, PPMS *n* = 1). First, it was examined to what extend GM-, WM- and CSF volume, sex and age at scan was driving variability in brain-PAD through hierarchical partitioning of variance. Altogether, an adjusted *R*^2^ *(AR*^2^*)* = 0.67 of the variance in brain-PAD was explained by the model. CSF (45.8%), GM (29.7%), WM (6.6%) and age at scan (17.9%) were independently contributing to this variance in brain-PAD whereas sex did not. As previous brain age studies have incorporated sex as covariate, statistical relevance for adjustment was implied and therefore sex was taken into account as a covariate (2, 3, 6). To avoid multi-collinearity, NBV was taken into account as a covariate along with age at scan and sex.

First, in bivariate analyses, higher Brain-PAD was found to be associated with both younger age at scanning (*r* = −0.16, *p* = 0.027) and younger age at onset of MS (*r* = −0.18, *p* = 0.011), whereas no correlation was found with disease duration (*p* = 0.22). Then, a linear regression model was performed with brain-PAD as independent variable. Brain-PAD was correlated with higher EDSS-score (*AR*^2^ = 0.206, β = 0.32, *p* < 0.01); higher 25-FWT-score (*AR*^2^ = 0.14, β = 0.47, *p* < 0.001); and higher 9 HPT-score (*AR*^2^ = 0.15, β = −0.24, *p* = 0.03) (Table 2). The correlation between brain-PAD and PASAT-score was not significant when adjusting for covariates. These results indicate an association between higher brain-PAD and worse physical but not cognitive performance on the PASAT. No influence of treatment on brain-PAD was found nor was there an influence of disease phase, even though brain-PAD was higher in the group of patients with enhanced lesions than without (mean brain-PAD: 5.4 and 9 respectively, *p* = 0.042). However, given that the presence of enhanced lesions did not explain any of the variance observed in brain-PAD, this was not taken further into account as a covariate.

**Table 2:** Standardized beta coefficients (β) from significant linear regression models of clinical and blood measures.

**Dependent variable**	**Brain-PAD**	**NBV**	**Age**	**Sex**
EDSS	0.32^**^	0.06	0.43^***^	−0.01
25-FWT	0.47^***^	0.27^*^	0.41^***^	0.05
Reciprocal 9-HPT	−0.24^*^	0.11	−0.02	−0.25^**^

*EDSS, expanded disability scale status; 25-FWT, 25-foot walk test; 9-HPT, reciprocal score of 9-hole peg test; Brain-PAD, brain predicted age difference; NBV, normalized brain volume*.

* *p < 0.05*,

** *p < 0.01*,

*** *p < 0.001*.

**Figure 2:**
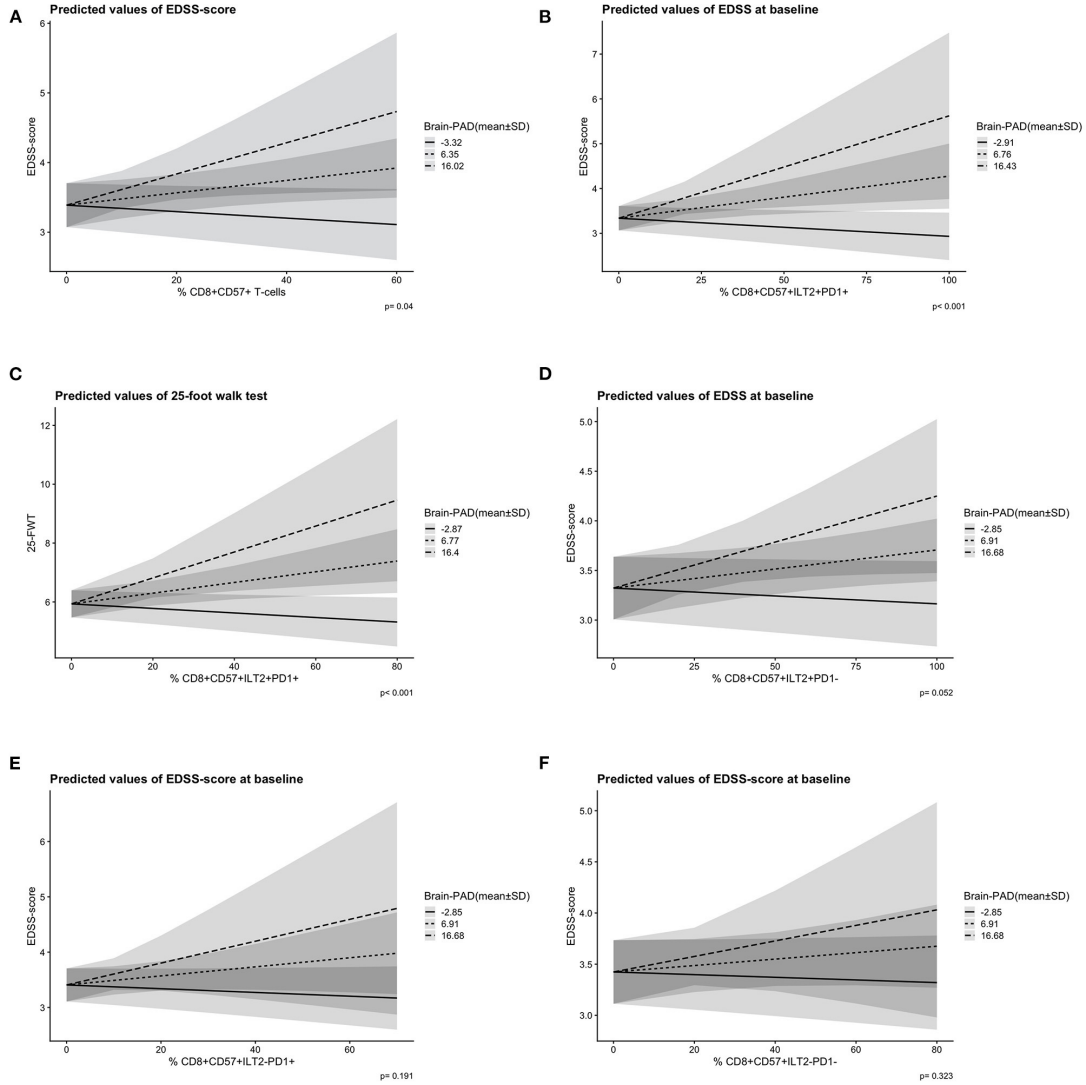
Predicted slopes illustrate the two-way interaction between ILT2/PD-1 positive and negative CD8^+^CD57^+^ T-cells and brain-PAD [mean ± 1 standard deviation (SD)] on disability score. **(A)** A higher CD8^+^CD57^+^ T-cell frequency (x-axis) and a higher brain-PAD (prediction lines) predicted a higher EDSS-score (y-axis) significantly, *p* = 0.04. **(B)** A higher CD8^+^CD57^+^ILT2^+^PD-1^+^ T-cell frequency (x-axis) and a higher brain-PAD (prediction lines) predicted a higher EDSS-score (y-axis) significantly, *p* < 0.001. **(C)** A higher CD8^+^CD57^+^ILT2^+^PD-1^+^ T-cell frequency (x-axis) and a higher brain-PAD (predicted lines) predicted a higher 25-FWT-score (y-axis) significantly, *p* < 0.001. **(D)** A non-significant interaction effect of CD8^+^CD57^+^ILT2^+^PD-1^−^ T-cells (x-axis) and brain-PAD (prediction lines) on EDSS-score (y-axis), *p* = 0.052. **(E)** A non-significant interaction effect of CD8^+^CD57^+^ILT2^−^PD-1^+^ T-cells (x-axis) and brain-PAD (prediction lines) on EDSS-score (y-axis), *p* = 0.191. **(F)** A non-significant interaction effect of CD8^+^CD57^+^ILT2^−^PD-1^−^ T-cells (x-axis) and brain-PAD (prediction lines) on EDSS-score (y-axis), *p* = 0.323. PD-1, programmed death 1; ILT2, immunoglobulin-like transcript 2; EDSS, expanded disability scale status; 25-FWT, 25-food walk test; brainPAD, brain predicted age difference (brain-PAD). Brain-PAD is divided in three subgroups based on the mean value of brain-PAD ± 1SD represented by a striped line (mean brain-PAD −1SD); a dotted line (mean brain-PAD); and a continuous line (mean brain-PAD + 1 SD). The shaded areas represent the 95% confidence intervals.

No significant correlation was seen between CD8^+^CD57^+^(ILT2^+^PD-1^+^) T-cell frequency and clinical disability measures (physical/cognitive), brain-PAD, chronological age or disease phase (*p* > 0.05). Treatment type, however, did have a significant influence on CD8^+^CD57^+^ILT2^−^PD-1^+^ T-cell frequency; a higher T-cell frequency was found in the patient group taking interferons (*n* = 49) compared to the off-treatment group (*n* = 99; mean difference = 6, *p* = 0.04). No independent predictive value of CD8^+^CD57^+^ T-cell level on disability score was found when combined with brain-PAD (*p* > 0.05). Yet, after adjusting for sex, age at scan and NBV, a higher EDSS-score was predicted by the interaction between a higher brain-PAD and a higher CD8^+^CD57^+^ T-cell frequency [*F*_(4,132)_ = 6.93, *p* = 0.041] (Figure 2A). In more detail, those who had a higher CD8^+^CD57^+^ILT2^+^PD-1^+^ T-cell frequency combined with a higher brain-PAD were predicted to have a higher EDSS-score [*F*_(4,139)_= 9.71, *p* < 0.001] (Figure 2B). A similar interaction effect was found when predicting 25-FWT-score [*F*_(4,121)_= 5.66, *p* < 0.001] (Figure 2C). However, AIC modeling showed no increase model accuracy when using the interaction term in the model instead of solely brain-PAD (brain-PAD *AR*^2^ = 0.206 vs. interaction brain-PAD and CD8^+^CD57^+^ILT2^+^PD-1^+^ T-cell *AR*^2^ = 0.196, ΔAICc = 1.8). The interaction effect between brain-PAD and CD8^+^CD57^+^ILT2^+^PD-1^−^ T-cell frequency on EDSS-score was close toward our predefined significance threshold of 0.05 (*p* = 0.052). No significant interaction effects were found when analyzing brain-PAD combined with the other PD-1 and ILT-2 CD8^+^CD57^+^ T-cell subtypes (Figures 2D–F).

## Discussion

In the current study, we examined whether the relationship of brain-PAD to disability in MS patients was influenced by the inflamm-aging T-cell biomarker CD8^+^CD57^+^. In accordance with previous research, we demonstrated that an older appearing brain was associated with higher physical disability in MS patients (2, 3, 23). No correlations were found between CD8^+^CD57^+^(ILT2^+^PD-1^+^) T-cell frequency and brain-PAD, or any demographical- or clinical variables apart from treatment group (13). Yet, an interaction between brain-PAD and CD8^+^CD57^+^(ILT2^+^PD-1^+^) T-cell frequency was found, suggesting that in MS patients with an older appearing brain, a higher number of these T-cells was associated with worse physical disability. Potentially, changes in the circulating inflammatory environment play a role in determining levels of physical disability in those MS patients who have experienced greater brain atrophy than would be expected for their age.

Contrary to our expectations we found no correlation between CD8^+^CD57^+^(ILT2^+^PD-1^+^) T-cell frequency and disability score. This could be due to several reasons. Firstly, this study was designed as observational study and not as a case-control study which precludes us to compare results with healthy controls. Moreover, no restrictions on drugs could be applied other than only including patients who were off corticosteroid therapy at least 3 months prior to enrolment, while in previous studies the intake of immunomodulatory drugs was paused two months prior to blood sampling (13, 24). The use of certain treatments can have a major impact on CD8^+^CD57^+^ T-cells, and thus could have affected the results (25). In our patient group, a higher cell frequency of CD8^+^CD57^+^ILT2^−^PD-1^+^ T-cells was found in the subgroup treated with interferons compared to the off-treatment group confirming an influence of the drugs on blood marker levels. This influence could have therefore altered any potential correlations between CD8^+^CD57^+^(ILT2^+^PD-1^+^) T-cell frequency and disability scores. However, due to a high variability in treatment type and duration prior registration, we should be cautious with drawing any conclusions about potential effects of treatments on the level of CD8^+^CD57^+^ILT2^−^PD-1^+^ T-cells in the peripheral blood based on this study.

Secondly, when assessing CD8^+^CD57^+^(ILT2^+^PD-1^+^) T-cell frequency, Cencioni et al. (13) found an increase of CD8^+^CD57^+^PD-1^+^ T-cells during quiescence (no relapse and no gadolinium-enhancing lesions present 3 months prior to blood sampling), while no such increase was found during relapse (relapse within 3 months prior to blood sampling). In the current study, such effect of disease phase on CD8^+^CD57^+^PD-1^+^ T-cell frequency was not found. This however, could be due to a difference in the used definition of disease phase. Where Cencioni et al. (13) focused on the presence of a relapse in the past 3 months, we defined our patients as active when having more than 2 relapses in the past year. Additionally, in the current cohort disease phase was unknown for 12% of our patients.

Earlier research found an increase in CD8^+^CD57^+^PD-1^+^ T-cells in the inflamed meninges of secondary progressive MS patients, suggesting an important role of these cells in MS progression (13). Moreover, it was shown that an upregulation of PD-1 expression by CD8^+^CD57^+^ T-cells was associated with a dysfunctional and reduced cytotoxic response upon virus infection, favoring a persistent inflammatory environment (13). Cencioni et al. (13) made the plausible suggestion that this increase in CD8^+^CD57^+^ T-cells may underly axonal damage leading to poorer structural brain health and disease progression in MS patients. As mentioned before, against our expectations, CD8^+^CD57^+^(PD-1^+^ILT2^+^) T-cell frequency did not relate to disability levels, suggesting that in a simple additive linear model, information on these inflammatory T-cells would not improve disability prediction by brain-PAD. However, the interaction found between CD8^+^CD57^+^ILT2^+^PD-1^+^ T-cell frequency and brain-PAD when predicting disability, suggests that in MS patients with a poorer brain health than expected for their age, CD8^+^CD57^+^ILT2^+^PD-1^+^ T-cell frequency may play a role in the development of physical disability. Additionally, the interaction between brain-PAD and CD8^+^CD57^+^ILT2^+^PD-1^−^ T-cell frequency was close toward significance when predicting EDSS-score, while the interaction between brain-PAD and CD8^+^CD57^+^ILT2^−^PD-1^+^ T-cell frequency was not. This finding confirmed the independent functioning of the ILT2 and PD-1 receptors on CD8^+^CD57^+^ T-cells which was previously suggested (13, 26). Gustafson et al. studied the cellular function of ILT2 and PD-1 in CD8^+^ T-cells during immune-aging and found that the major function of ILT2 was constraining proliferation, while unlike PD-1, leaving cytotoxic CD8^+^CD57^+^ T-cell functioning upon virus infection intact (27). This, together with our findings, suggests that ILT2 might complement the inhibiting effect of PD-1 on CD8^+^CD57^+^ T-cells in an analog but unique way. More research should be done to ILT2 and PD-1 functioning on CD8^+^CD57^+^ T-cells to gain more in-depth knowledge about its role in physical disability development in MS patients.

This study provided us with the unique opportunity to gain insights into the biological mechanisms underlying MS disease progression and gain knowledge about how these mechanisms relate to the biology of aging by combining measures of cellular senescence with measures of brain structure. Our results need validation in larger cohorts, and would benefit from comparison with healthy controls to ascertain the specificity of the observed relationships to MS. In future research, the patient's disease phase (relapsing or stable) should be logged carefully, given the distinctive results for both groups found by Cencioni et al. (13). To avoid the interference of drug use with blood test results, only patients who did not use immunomodulatory drugs 2 months prior blood sampling should be included. With these conditions in mind, a longitudinal multi-center MS study could be conducted, in which brain age is examined over time and combined with the CD8^+^CD57^+^(PD-1^+^ILT2^+^) T-cell activity and another aging biomarker such as the epigenetic clock, derived from DNA methylation patterns (6). Lastly, multiple disability scores should be collected over time to avoid dependency on EDSS-score for cross-sectional and longitudinal analysis.

We provide further evidence that the brain age paradigm is sensitive to physical disability in MS. Moreover, a higher number of CD8^+^CD57^+^ILT2^+^PD-1^+^ T-cells in the PB in patients with an older appearing brain was related to disability scores. This finding suggests that CD8^+^CD57^+^ILT2^+^PD-1^+^ T-cells may play a role in the development of physical disability in MS patients who have poorer brain health (i.e., greater atrophy than expected for their age). Future research is needed to establish the utility of brain age as a prognostic marker combined with CD8^+^CD57^+^ILT2^+^PD-1^+^ T-cells in a multi-center longitudinal study design including healthy controls.

## Data Availability Statement

The datasets presented in this article are not readily available because they contain information that could compromise the privacy of research participants. Therefore, only the linked anonymized data will be available outside the patients treating teams. When sharing, data will be managed according to the trial's ethical permissions. Requests to access the anonymized datasets should be directed to Richard Nicholas, r.nicholas@imperial.ac.uk.

## Ethics Statement

The studies involving human participants were reviewed and approved by London-Chelsea Regional Ethics Committee (NCT01044576). The patients/participants provided their written informed consent to participate in this study.

## Author Contributions

SJ conducted the statistical analyses and writing. Together with SJ and SK analyzed T-1 weighted MRI images with the brainageR software. PM collected this MRI data. MC collected the immunological data. RN collected the clinical data. RN and JC supported SJ in writing and statistical analyses. JC designed the brainageR software. All authors contributed to the article and approved the submitted version.

## Funding

This work was supported by funding from Medical Research Council (to PM, Ref. No. G0800679), from the NIHR Biomedical Research Centre funding scheme and was carried out in part at the NIHR/Welcome Trust Imperial Clinical Research Facility at Hammersmith Hospital, London. We also acknowledge support to MC from the Elena Pecci research project and the Fondazione Careggi Onlus.

## Author Disclaimer

The views expressed are those of the authors and not necessarily those of the MRC, the NHS, the NIHR or the Department of Health.

## Conflict of Interest

JC is an advisor to and shareholder in BrainKey and Claristas HealthTech, and acts as a consultant to Queen Square Analytics. PM declares no conflicting interest relevant to this study, but discloses travel support and speaker honoraria from unrestricted educational activities organized by Novartis, Bayer HealthCare, Bayer Pharma, Biogen Idec, Merck-Serono and Sanofi Aventis; and consulting to Magenta Therapeutics and Jasper Therapeutics. RN has received funding for paid advisory boards with Roche, Novartis, Biogen and ImmunBio and has participated in trials run by Roche, Novartis and Biogen. The remaining authors declare that the research was conducted in the absence of any commercial or financial relationships that could be construed as a potential conflict of interest.

## Publisher's Note

All claims expressed in this article are solely those of the authors and do not necessarily represent those of their affiliated organizations, or those of the publisher, the editors and the reviewers. Any product that may be evaluated in this article, or claim that may be made by its manufacturer, is not guaranteed or endorsed by the publisher.
